# Supplementary material for: Robust detection of clinically relevant features in single-cell RNA profiles of patient-matched fresh and formalin-fixed paraffin-embedded (FFPE) lung cancer tissue
Source: Cell Oncol (Dordr). 2024 Feb 1;47(4):1221–31. doi: 10.1007/s13402-024-00922-0 (PMC11322409; doi:10.1007/s13402-024-00922-0)
Supplement: Supplementary file 1 — Supplementary Material 1 [file 13402_2024_922_MOESM1_ESM.pdf]

Supplementary figures

Robust detection of clinically relevant features in single-cell RNA profiles of patient-matched fresh and formalin-fixed paraffin-embedded (FFPE) lung cancer tissue

Alexandra Trinks[1], Miha Milek[2], Dieter Beule[2], Julie Kluge[3], Stefan Florian[3,4], Christine Sers[3,5], David Horst[3,5], Markus Morkel[1,3,5,*], and Philip Bischoff[3,4,5,*]

Affiliations:

1) Berlin Institute of Health at Charité – Universitätsmedizin Berlin, Bioportal Single Cells, Berlin, Germany
2) Berlin Institute of Health at Charité – Universitätsmedizin Berlin, Core Unit Bioinformatics (CUBI), Berlin, Germany
3) Charité – Universitätsmedizin Berlin, Corporate Member of Freie Universität Berlin and Humboldt-Universität zu Berlin, Institute of Pathology, Berlin, Germany
4) Berlin Institute of Health at Charité – Universitätsmedizin Berlin, BIH Biomedical Innovation Academy, BIH Charité Clinician Scientist Program, Berlin, Germany
5) German Cancer Consortium (DKTK) Partner Site Berlin, German Cancer Research Center (DKFZ), Heidelberg, Germany

* equal contribution

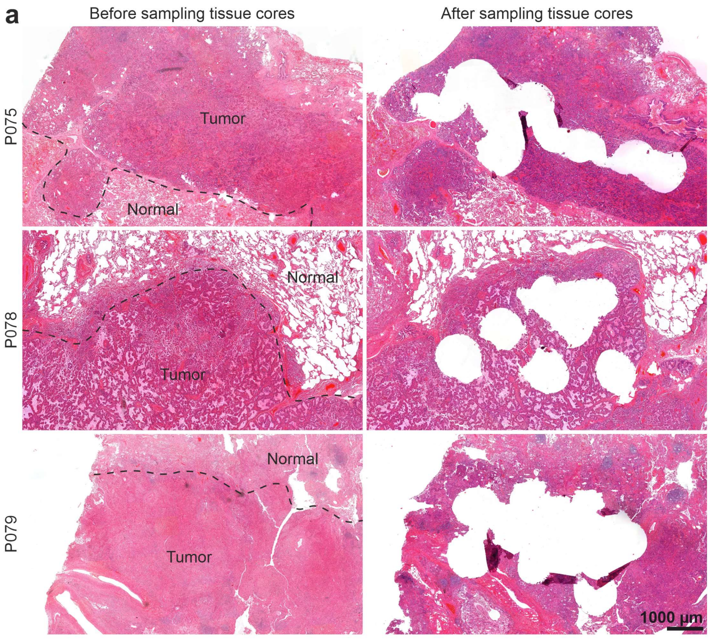

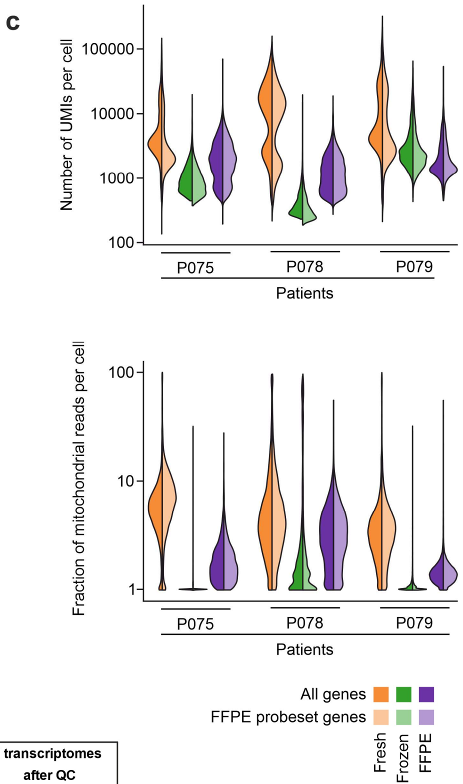

b

Samples			Number of cells/nuclei in suspension					Number of single-cell transcriptomes	
Patient ID	Sample type	Run No.	used for hybridisation	after FACS	before loading Chromium chip	loaded onto Chromium chip	targeted after Chromium run	before QC filtering	after QC filtering
P075	Fresh	1	NA	NA	600k (1,200/ul)	16.5k	10k	8,147	7,876
P078	Fresh	1	NA	NA	550k (1,100/ul)	16.5k	10k	8,320	7,703
P079	Fresh	1	NA	NA	600k (1,200/ul)	16.5k	10k	8,942	8,600
P075	FFPE	1	5000k	200k	48k (600/ul)	16.5k	10k	9,044	8,844
P078	FFPE	1	500k	177k	72k (900/ul)	16.5k	10k	7,024	6,468
P079	FFPE	1	2000k	200k	48k (600/ul)	16.5k	10k	8,927	8,890
P075	Frozen	1	NA	200k	40k (500/ul)	16.5k	10k	9,197	9,016
P078	Frozen	1	NA	200k	40k (500/ul)	16.5k	10k	42,076	41,782
P079	Frozen	1	NA	200k	40k (500/ul)	16.5k	10k	34,465	34,293
P075	Frozen	2	NA	200k	48k (600/ul)	16.5k	10k	8,827	8,510
P078	Frozen	2	NA	200k	48k (600/ul)	16.5k	10k	7,686	1,478
P079	Frozen	2	NA	200k	48k (600/ul)	16.5k	10k	4,671	4,667

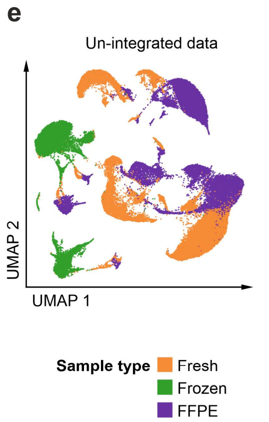

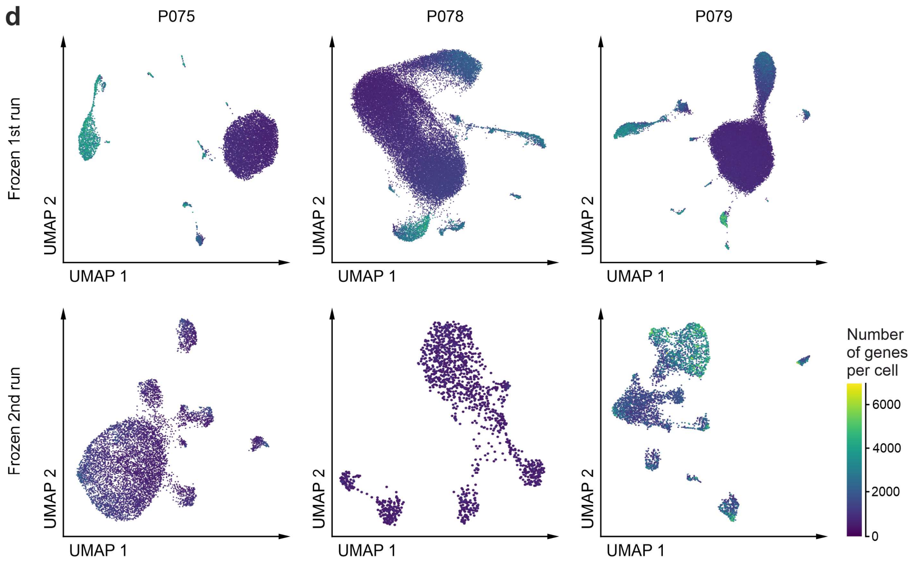

Figure S1: Additional quality metrics of fresh, frozen and FFPE tissue.

(a) Hematoxylin and eosin stained FFPE tissue sections of patients P075, P078 and P079 before and after punching out tissue core samples for single-cell analysis, tumor and normal areas

indicated. **(b)** Total numbers of cells/nuclei and single-cell transcriptomes at different steps in tissue processing and data analysis. **(c)** Numbers of unique molecule identifiers (UMIs) and fractions of mitochondrial reads per cell across libraries. Full colors: all genes; lighter colors: genes limited by FFPE probe set. **(d)** UMAPs of frozen tissue libraries showing insufficient cluster separation. Only patient P079, 2nd run shows sufficient cluster separation, and thus cell type annotation. **(c)** UMAPs based on the top 10 principal components of all single-cell transcriptomes after filtering and normalization, without data integration, color-coded by fresh, frozen or FFPE tissue origin. For UMAP of integrated data, see main Fig. 1C.

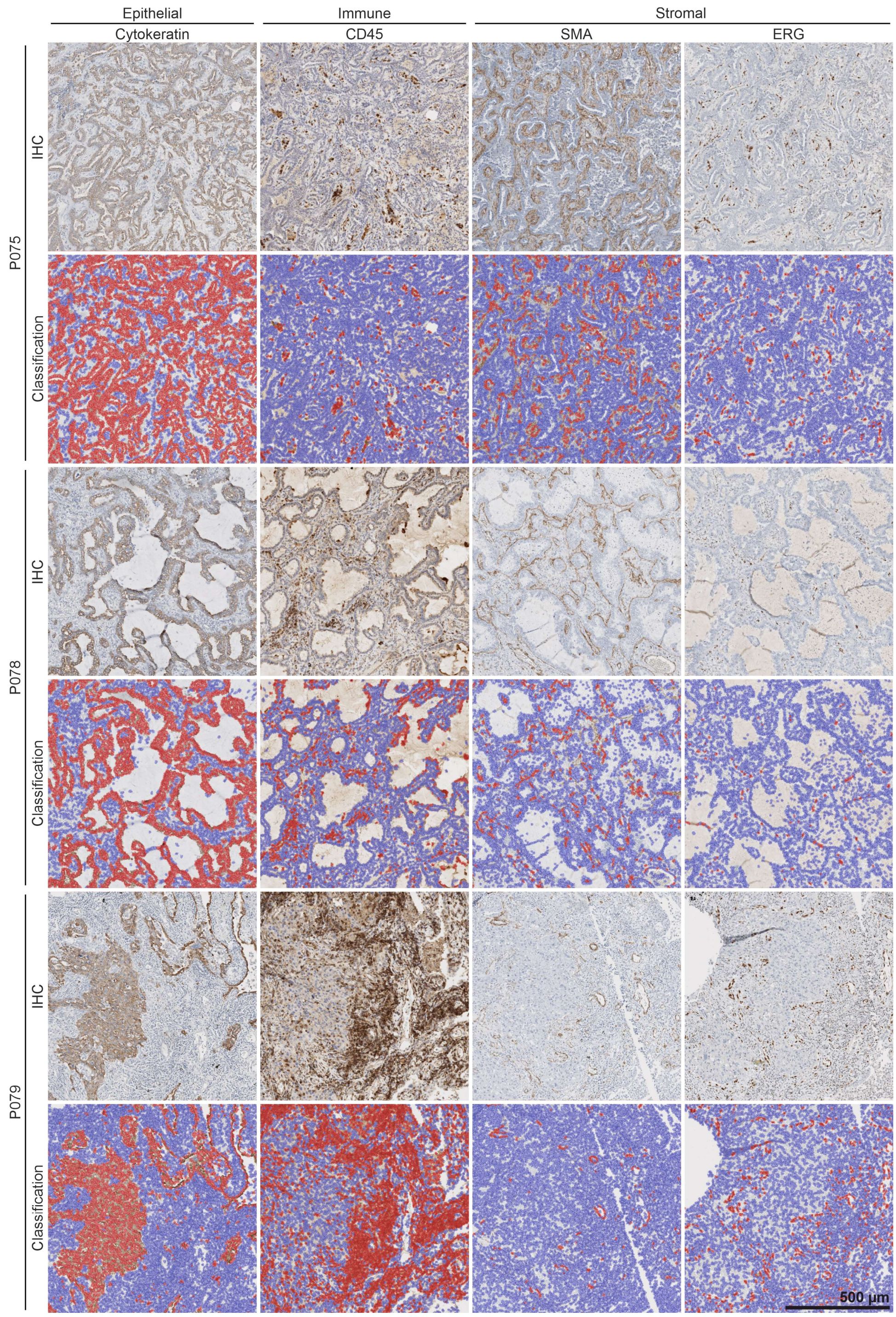

Figure S2: Immunohistochemical quantification of main cell types.

Immunohistochemistry (IHC) on FFPE tumor sections for main cell type markers, including Cytokeratin for epithelial cells, CD45 for immune cells, and Smooth Muscle Actin (SMA) and ERG for stromal cells, representative images shown. For quantification, cells were detected based on nuclei counterstaining and classified into positive (red) and negative (blue) cells based on IHC intensity using the QuPath software, classified images depicted below respective IHC images.

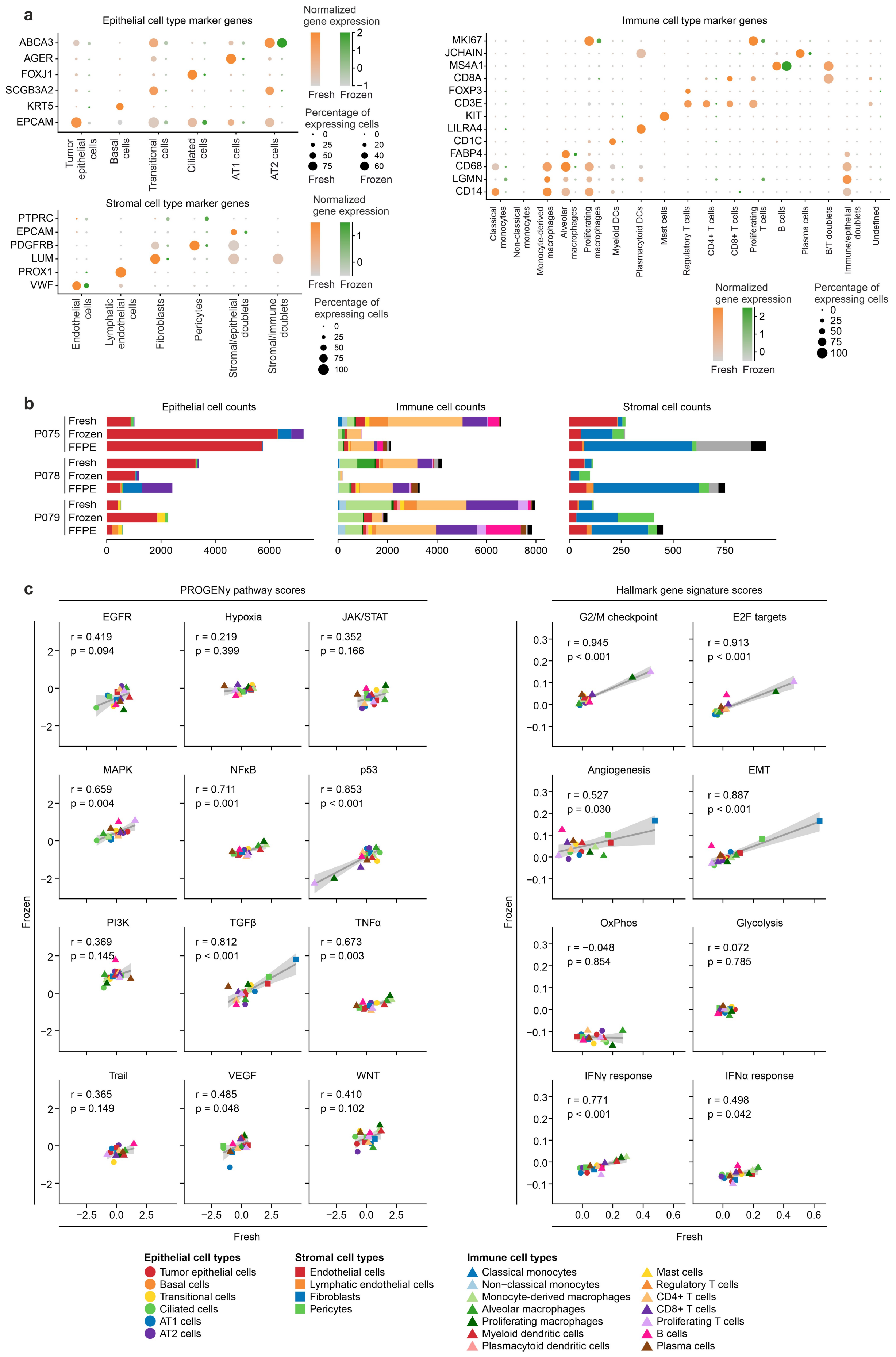
a
Epithelial cell type marker genes
ABCA3
AGER
FOXJ1
SCGB3A2
KRT5
EPCAM
Tumor epithelial cells
Basal cells
Transitional cells
Ciliated cells
AT1 cells
AT2 cells
Normalized gene expression
Fresh Frozen
Percentage of expressing cells
Stromal cell type marker genes
PTPRC
EPCAM
PDGFRB
LUM
PROX1
VWF
Endothelial cells
Lymphatic endothelial cells
Fibroblasts
Pericytes
Stromal/epithelial doublets
Stromal/immune doublets
Immune cell type marker genes
MKI67
JCHAIN
MS4A1
CD8A
FOXP3
CD3E
KIT
LILRA4
CD1C
FABP4
CD68
LGMN
CD14
Classical monocytes
Non-classical monocytes
Monocyte-derived macrophages
Alveolar macrophages
Proliferating macrophages
Myeloid DCs
Plasmacytoid DCs
Mast cells
Regulatory T cells
CD4+ T cells
CD8+ T cells
Proliferating T cells
B cells
Plasma cells
B/T doublets
Immune/epithelial doublets
Undefined
b
Epithelial cell counts
Immune cell counts
Stromal cell counts
P075
P078
P079
Fresh
Frozen
FFPE
c
PROGENy pathway scores
EGFR
r = 0.419
p = 0.094
Hypoxia
r = 0.219
p = 0.399
JAK/STAT
r = 0.352
p = 0.166
MAPK
r = 0.659
p = 0.004
NFκB
r = 0.711
p = 0.001
p53
r = 0.853
p < 0.001
PI3K
r = 0.369
p = 0.145
TGFβ
r = 0.812
p < 0.001
TNFα
r = 0.673
p = 0.003
Trail
r = 0.365
p = 0.149
VEGF
r = 0.485
p = 0.048
WNT
r = 0.410
p = 0.102
Hallmark gene signature scores
G2/M checkpoint
r = 0.945
p < 0.001
E2F targets
r = 0.913
p < 0.001
Angiogenesis
r = 0.527
p = 0.030
EMT
r = 0.887
p < 0.001
OxPhos
r = −0.048
p = 0.854
Glycolysis
r = 0.072
p = 0.785
IFNγ response
r = 0.771
p < 0.001
IFNα response
r = 0.498
p = 0.042
Frozen
Fresh
Epithelial cell types
Tumor epithelial cells
Basal cells
Transitional cells
Ciliated cells
AT1 cells
AT2 cells
Stromal cell types
Endothelial cells
Lymphatic endothelial cells
Fibroblasts
Pericytes
Immune cell types
Classical monocytes
Non-classical monocytes
Monocyte-derived macrophages
Alveolar macrophages
Proliferating macrophages
Myeloid dendritic cells
Plasmacytoid dendritic cells
Mast cells
Regulatory T cells
CD4+ T cells
CD8+ T cells
Proliferating T cells
B cells
Plasma cells

Figure S3: Cell type diversity in fresh, frozen and FFPE tissue single-cell analysis.

(a) Cell type marker gene expression per cell type in fresh or frozen tissue-derived libraries. **(b)** Absolute cell numbers per cell type, in fresh, frozen or FFPE-derived libraries. **(c)** Cell trait quantification in fresh versus frozen single-cell analysis. Correlations of PROGENy pathway or Hallmark signature scores are given between frozen and fresh tissue gene expression per cell type. Pearson correlation coefficient and p-value indicated per pathway or gene signature.

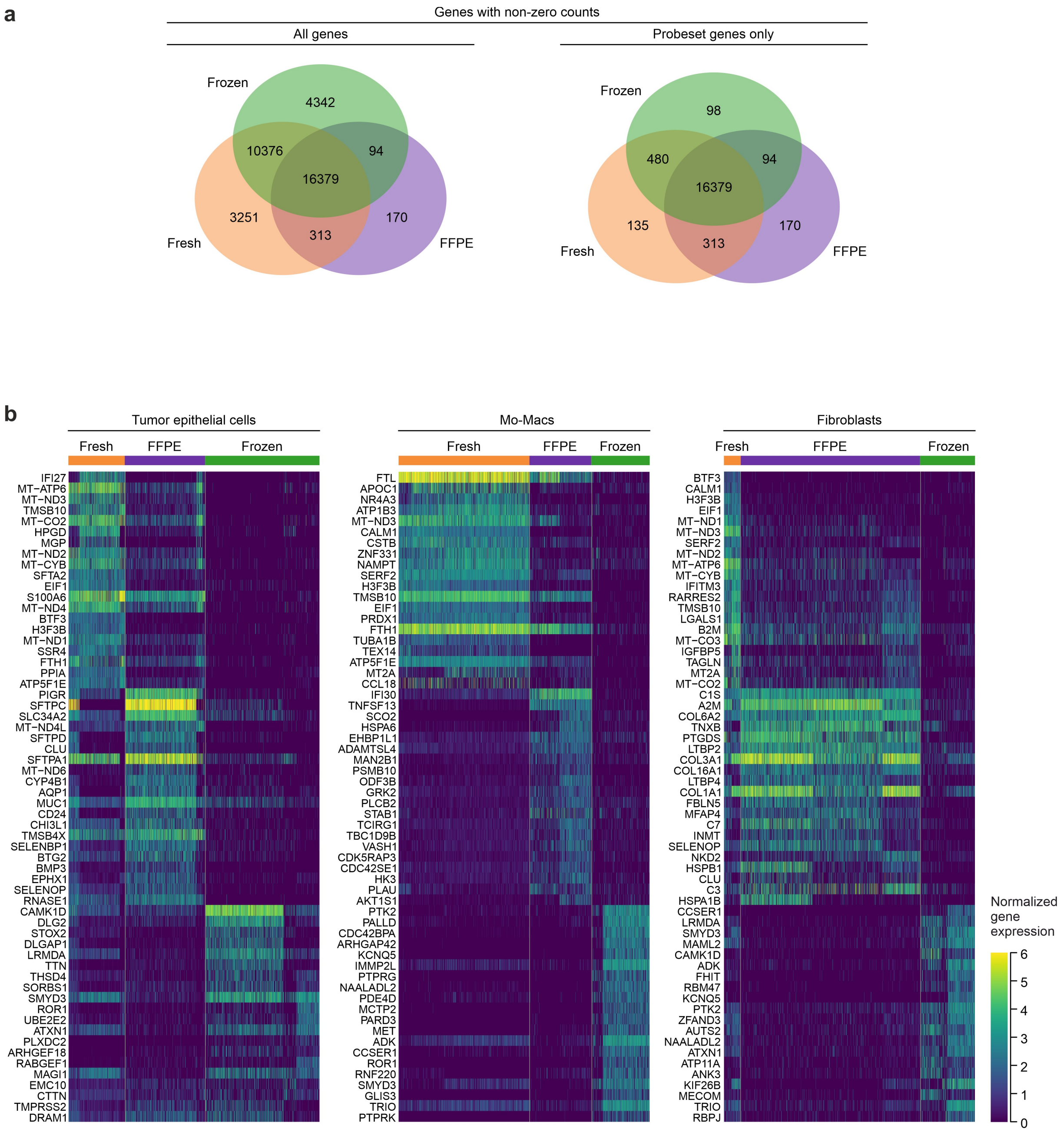

Figure S4: Gene expression quantification across single-cell libraries.

(a) Numbers of genes detected (non-zero counts) across the technologies. To the left: all genes, to the right: genes represented by FFPE probe sets only. **(b)** Top 20 differentially expressed genes across the technologies in tumor epithelial cells, monocyte-derived macrophages (Mo-Macs) and fibroblasts, respectively.

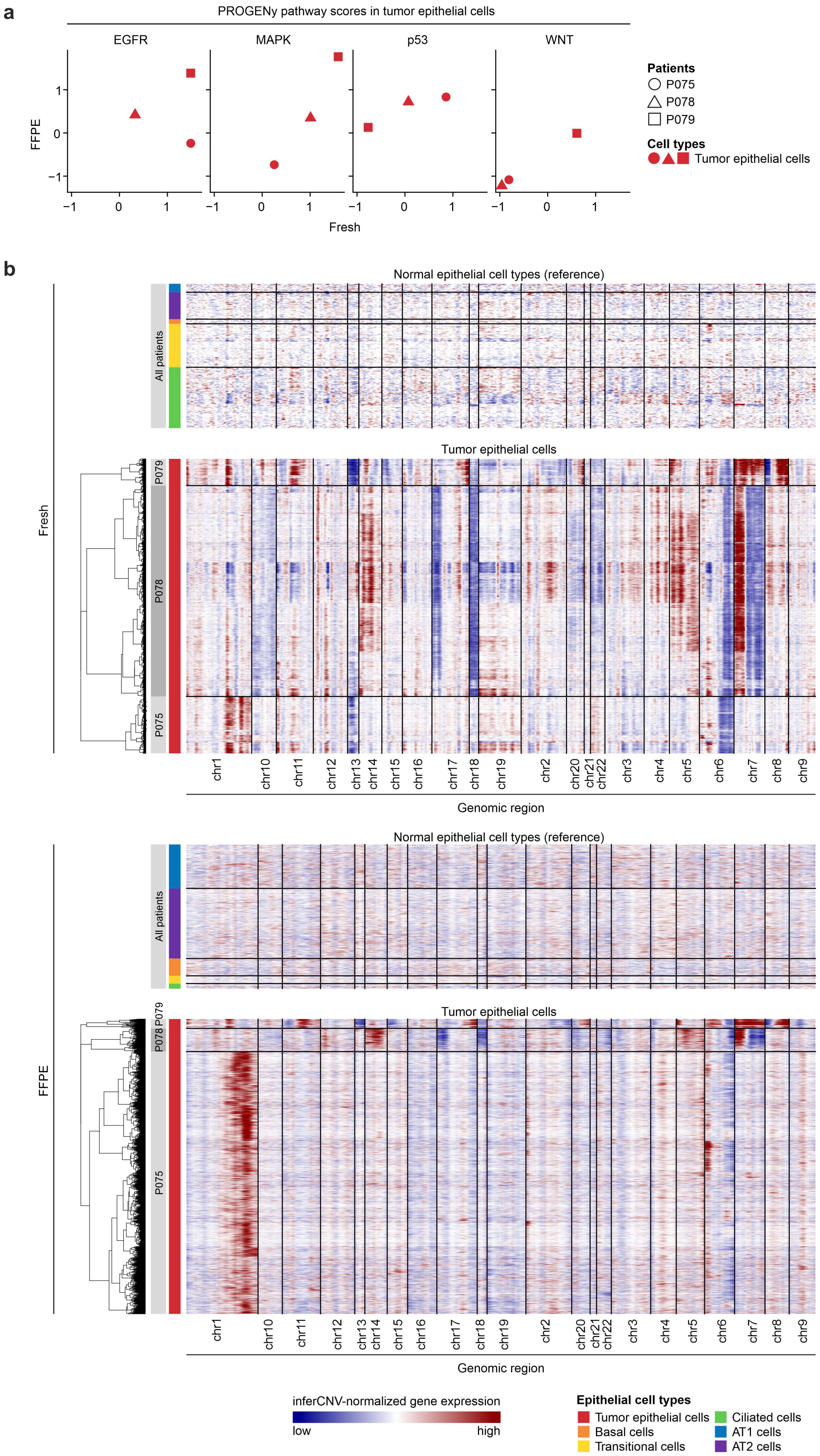
a
PROGENy pathway scores in tumor epithelial cells
EGFR
MAPK
p53
WNT
FFPE
Fresh
Patients
P075
P078
P079
Cell types
Tumor epithelial cells
b
Normal epithelial cell types (reference)
Tumor epithelial cells
All patients
Fresh
FFPE
P079
P078
P075
chr1
chr10
chr11
chr12
chr13
chr14
chr15
chr16
chr17
chr18
chr19
chr2
chr20
chr21
chr22
chr3
chr4
chr5
chr6
chr7
chr8
chr9
Genomic region
inferCNV-normalized gene expression
low
high
Epithelial cell types
Tumor epithelial cells
Basal cells
Transitional cells
Ciliated cells
AT1 cells
AT2 cells

Figure S5: Quantification of oncogenic pathway activities and inference of copy number aberrations in fresh versus FFPE single-cell analysis of tumor epithelial cells.

(a) Correlation between FFPE and fresh tissue of PROGENy EGFR, MAPK, p53 and Wnt target gene signatures, as indicated. **(b)** Predicted copy number aberrations inferred from tumor epithelial single-cell transcriptomes of fresh or FFPE tissue origin, as indicated. Normal epithelial cells served as reference. In fresh and FFPE tissue samples, shared copy number aberrations were observed, such as partial gain of chromosome 1 in P075, loss of chromosome 18 and partial loss of chromosome 17 in P078, and gain of chromosome 7 and loss of chromosome 13 in P079.
