# Supplementary material for: Robust detection of clinically relevant features in single-cell RNA profiles of patient-matched fresh and formalin-fixed paraffin-embedded (FFPE) lung cancer tissue
Source: Cell Oncol (Dordr). 2024 Feb 1;47(4):1221–31. doi: 10.1007/s13402-024-00922-0 (PMC11322409; doi:10.1007/s13402-024-00922-0)
Supplement: Supplementary file 2 — Supplementary Material 2 [file 13402_2024_922_MOESM2_ESM.pdf]

Supplementary Method: Processing of FFPE tissue samples for single-nuclei RNA sequencing

Robust detection of clinically relevant features in single-cell RNA profiles of patient-matched fresh and formalin-fixed paraffin-embedded (FFPE) lung cancer tissue

Alexandra Trinks[1], Miha Milek[2], Dieter Beule[2], Julie Kluge[3], Stefan Florian[3,4], Christine Sers[3,5], David Horst[3,5], Markus Morkel[1,3,5,*], and Philip Bischoff[3,4,5,*]

Affiliations:
1) Berlin Institute of Health at Charité – Universitätsmedizin Berlin, Bioportal Single Cells, Berlin, Germany
2) Berlin Institute of Health at Charité – Universitätsmedizin Berlin, Core Unit Bioinformatics (CUBI), Berlin, Germany
3) Charité – Universitätsmedizin Berlin, Corporate Member of Freie Universität Berlin and Humboldt-Universität zu Berlin, Institute of Pathology, Berlin, Germany
4) Berlin Institute of Health at Charité – Universitätsmedizin Berlin, BIH Biomedical Innovation Academy, BIH Charité Clinician Scientist Program, Berlin, Germany
5) German Cancer Consortium (DKTK) Partner Site Berlin, German Cancer Research Center (DKFZ), Heidelberg, Germany

* equal contribution

Preface:
This protocol is based on the demonstrated protocol “Isolating Cells from FFPE Tissue Sections for Chromium Fixed RNA Profiling” version CG000606_Rev B from 10X Genomics. Additional modifications have been adopted from Vallejo, A. F. et al. “snPATHO-seq: unlocking the FFPE archives for single nucleus RNA profiling.” bioRxiv 2022.08.23.505054 (2022) doi:10.1101/2022.08.23.505054. These include passing the sample through a 25G needle during dissociation and the optional FACS sorting step for debris removal.

1. Materials and Reagents

Equipment/Material	Company
S2 sterile hood	Any vendor
FACS Cell Sorter	BD Aria or equivalent
Flourescent Microscope	Any vendor
Microtom (for FFPE slices)	Any vendor
Biopsy punch 1mm (for FFPE punches)	Pfm medical, 49101
Pipets	Any vendor
Pipet tips (10, 200, 1000µl)	Any vendor
GentleMACS Dissociator	MACS, 130-096-427
1.5ml Benchtop-Centrifuge	Eppendorf, 5415R or equivalent
15/50ml Centrifuge	Heraeus, Multifuge X3R or equivalent
Neubauer Cell Count Chamber	Any model
Heatblock at 42C, 1.5ml adapter	Any vendor
Heatblock at 65C, 2ml adapter	Any vendor
Petri dish (5cm)	Corning Life Science or equivalent
Reaction tubes (1.5ml)	Eppendorf LoBind, 0030108051
Falcon tubes (50 ml)	Corning Life Science or equivalent
Miltenyi C tubes (50ml)	Miltenyi, 130-096-334
Miltenyi Cell strainer (100µm) for 1.5ml	Miltenyi, 130-110-917
Miltenyi Cell strainer (20µm) for 1.5ml	Miltenyi, 130-101-812
Cell strainer (70um) for 50ml	Corning, 431751
Cell strainer (40um) for 50ml	Corning, 431750
Scalpel	Any vendor
Forceps	Any vendor
Serological Pipettes	Any vendor
Filter 0.22uM (for filtering buffers)	Sarstedt, 83.1826.001
Syringe 20ml	BD Plastipak, 300629
20G or 25G needle	Sterican, 4665791
Round bottom tube with cell strainer cap	Becton Dickinson, 352235
Ice Box	Any vendor
Cool Rack, 1.5ml, 0.2ml	Any vendor

Reagent	Company
Miltenyi FFPE kit, human	Miltenyi, 130-118-052
Xylene	Sigma Aldrich, 214736
Ethanol absolute, 99%	Merck, 11093.03
1x PBS (1xDPBS, w/o CaCl2, MgCl2)	Gibco, Thermo Fisher, #14190-094
dH2O	Biozym, 331705
100% BSA (Albumin bovine Fraction V)	Serva, #11930
BSA, 10% Aqueous Solution, Nuc-Free	Sigma Aldrich, 126615
Tris, pH 8	VWR, E199-100ML
RNase Inhibitor, cloned (40U µL-1)	Ambion, AM2682
DAPI Staining Solution	Miltenyi, 130-111-570
50% Glycerol	BioVision, B1012-100
SPRIselect beads	Beckman Coulter, B23318
10% Tween 20	Roche, 11332465001
10X 3’scRNA Flex kit + Chip Q	10X Genomics, diverse

2. Reagent Preparation

Miltenyi FFPE Kit

Enzyme D (~ 6 months at -20C)

- dissolve in 3ml sterile H2O, invert during 5-10min (no vortex)
- make 200ul aliquots
- store at -20C

Enzyme G (~ 6 months at -20C)

- dissolve in 3ml sterile H2O, invert during 5-10min (no vortex)
- make 200ul aliquots
- store at -20C

3. Buffer Preparation

De-paraffining per sample

Reagent	Stock Conc	Final Conc	Reagent (ml)	H2O (ml)	Final Volume
Xylene	100%	100%	9ml	-	9ml
Ethanol	100%	100%	4ml	-	4ml
Ethanol	100%	70%	0.7ml	0.3ml	1ml
Ethanol	100%	50%	0.5ml	0.5ml	1ml
H2O	100%	100%	1ml	-	1ml
Buffer V	100%	100%	1ml	-	1ml

Dissociation Buffer 2.5ml per sample (Miltenyi FFPE kit)

Reagent	Stock Conc	Final Conc	Volume 1x	Volume 4x	Volume 8x
Buffer V	-	-	2.3ml	9.2ml	18.4ml
Enzyme D	-	-	100ul	400ul	800ul
Enzyme G	-	-	100ul	400ul	800ul
Reagent A	-	-	2.5ul	10ul	20ul

Resuspension Buffer 500ul per sample

Reagent	Stock Conc	Final Conc	Volume 1x	Volume 4x	Volume 8x
PBS	1x	0.496x	248ul	992ul	1984ul
Tris pH8	1M	50mM	25ul	100ul	200ul
BSA	10%	0.02%	1ul	4ul	8ul
RNase Inh	40U/ul	0.24U/ul	3ul	12ul	24ul
H_2O	-	-	223ul	892ul	1784ul

10X Hyb-Mix 80ul per sample + 20ul individual barcode

Reagent	Temperature	Volume 1x	Volume 4x	Volume 8x
Hyb Buffer B	Thaw at 42C, keep warm	70ul	280ul	560
Enhancer	Thaw at 65C, keep at 42C	10ul	40ul	80ul
Incubate mix	5min at 42C	80ul/sample	80ul/sample	80ul/sample
		**+individual**	**+individual**	**+individual**
WTA Probes	Thaw on ice, vortex	20ul	20ul	20ul

10X Post-Hyb Wash Buffer 1.75ml per sample

Reagent	Temperature	Volume 1x	Volume 4x	Volume 8x
dH2O	RT	1.98ml	6.93ml	13.86ml
Conc Buffer	Thaw at RT, keep on ice	110ul	385ul	770ul
Enhancer	Thaw at 65C, keep at 42C	110ul	385ul	770ul

10X Post-Hyb Resuspension Buffer 500ul for FACS

Reagent	Temperature	Volume 1x
dH2O	RT	523ul
Conc Buffer	Thaw at RT, keep on ice	27.5ul

4. Procedure

Stage 1: FFPE Sample Collection

- Select target area on FFPE block (ideally cell-rich) through inspection by a pathologist

Punches:

- Use a biopsy punch to collect 1mm x 3mm punches in a petri dish
- Remove visible paraffin and cut punches in smaller pieces using a scalpel
- Transfer into a Miltenyi C-Tube

Slices:

- Cut 50um thick slices using a microtome into a Miltenyi C-Tube
- Number of slices depend on cell-richness

Stage 2: De-paraffinization

- Add the following reagents to the sample, incubate at RT, remove

Miltenyi C-Tube
3ml Xylene – 10min
3ml Xylene – 10min
3ml Xylene – 10min
3ml Ethanol 100% – 1min
1ml Ethanol 100% – 1min
1ml Ethanol 70% – 1min
1ml Ethanol 50% – 1min
**Make sure paraffin is removed**
1ml dH2O – 1min
0.5ml Buffer V – 1min

Stage 3: Nuclei Isolation

- Prepare Dissociation Buffer and add 2.5ml to Miltenyi C-Tube

Dissociation Buffer 2.5ml per sample (Miltenyi FFPE kit)

Reagent	Stock Conc	Final Conc	Volume 1x	Volume 4x	Volume 8x
Buffer V	-	-	2.3ml	9.2ml	18.4ml
Enzyme D	-	-	100ul	400ul	800ul
Enzyme G	-	-	100ul	400ul	800ul
Reagent A	-	-	2.5ul	10ul	20ul

Miltenyi C-Tube
**GentleMacs**
Run FFPE program with heaters

Turn on heat-blocks: 2ml block at 65C, 1.5ml block at 42C

- Quick spin
- When solution is cloudy and punches are dissolved, use a 25G needle 10-15x to rupture tissue further into single cells; be gentle, avoid bubbles
- Pass solution through a 70um filter into a 50ml falcon
- Wash tube and filter with 1ml Buffer W
- Check nuclei under microscope and count; aim for 0.5-2million nuclei per sample
- Spin 5min at 850g at RT
- Remove supernatant without touching the pellet and resuspend in 500ul Resuspension Buffer

Resuspension Buffer 500ul per sample

Reagent	Stock Conc	Final Conc	Volume 1x	Volume 4x	Volume 8x
PBS	1x	0.496x	248ul	992ul	1984ul
Tris pH8	1M	50mM	25ul	100ul	200ul
BSA	10%	0.02%	1ul	4ul	8ul
RNase Inh	40U/ul	0.24U/ul	3ul	12ul	24ul
H_2O	-	-	223ul	892ul	1784ul

- Pass solution through a 20um filter (no flow-mi) into a low bind 1.5ml tube
- Spin 5min at 850g at RT

Stage 4: Probe Hybridization

- Heat up reagents and prepare 10X Hyb-Mix, incubate mix for 5min at 42C
- Thaw WTA Probes on ice, vortex, spin

10X Hyb-Mix 80ul per sample + 20ul individual barcode

Reagent	Temperature	Volume 1x	Volume 4x	Volume 8x
Hyb Buffer B	Thaw at 42C, keep warm	70ul	280ul	560
Enhancer	10min at 65C, keep at 42C	10ul	40ul	80ul
Incubate mix	5min at 42C	80ul/sample	80ul/sample	80ul/sample
		**+individual**	**+individual**	**+individual**
WTA Probes	Thaw on ice, vortex, spin	20ul	20ul	20ul

- Remove supernatant without touching the pellet and resuspend in 80ul 10X Hyb-Mix
- Add 20ul of individual WTA Probe
- Incubate 16-24h at 42C without shaking

Stage 5: Post Hybridization Wash

- Prepare reagents

Reagent	Temperature
TL v1 Gel Beads	Thaw at RT, vortex 30sec
Reducing Agent	Thaw at RT, vortex, spin
Conc Post Hyb Buffer	Thaw at RT, keep on ice
GEM Reagent Mix	Thaw at RT, keep on ice, vortex, spin
GEM Enzyme Mix	Keep at -20C until use, spin
Enhancer	Thaw at 65C, vortex, keep at 42C
Partitioning Oil	RT
50% Glycerol	RT

- Prepare Post Hyb Wash Buffer and Post Hyb Resuspension Buffer

10X Post-Hyb Wash Buffer 1.75ml per sample

Reagent	Temperature	Volume 1x	Volume 4x	Volume 8x
dH2O	RT	1.98ml	6.93ml	13.86ml
Conc Buffer	Thaw at RT, keep on ice	110ul	385ul	770ul
Enhancer	Thaw at 65C, keep at 42C	110ul	385ul	770ul

10X Post-Hyb Resuspension Buffer 500ul for FACS

Reagent	Temperature	Volume 1x
dH2O	RT	523ul
Conc Buffer	Thaw at RT, keep on ice	27.5ul

- Add 750ul Post Hyb Wash Buffer to each sample
- Incubate 10min at 42C
- Spin 5min at 850g at RT
- Remove supernatant
- Add 500ul Post Hyb Wash Buffer to each sample
- Incubate 10min at 42C
- Spin 5min at 850g at RT
- Remove supernatant
- Add 500ul Post Hyb Wash Buffer to each sample
- Incubate 10min at 42C
- Transfer sample over a filter cap into a FACS tube

Stage 6: FACS sort & Multiplexing

- Proceed immediately to a FACS sorter

Reagent	Temperature
Sample in FACS tube	On ice
DAPI	On ice
Post Hyb Resuspension Buffer	On ice
LB 1.5ml tube to facs sort into	RT

- Record each unstained sample
- Add 100ul Post Hyb Resuspension Buffer into LB 1.5ml tube for sample collection
- Stain each sample with 5ul DAPI solution, incubate a few minutes on ice
- Start sorting of DAPI-positive nuclei
- After sort, spin each collection tube 10min at 850g at 4C
- Remove supernatant, leave ~80ul behind

Stage 7: Chromium loading

- Proceed with 10X User Guide, CG000691
- Count nuclei and determine concentration according to 10X loading table
- Prepare 10X MasterMix and Chip for Chromium run and proceed with library prep according to manufacturer's protocol
